# Tumor Necrosis Factor-Superfamily 15 Gene Expression in Patients with Sickle Cell Disease

**DOI:** 10.4274/tjh.2012.0130

**Published:** 2014-09-05

**Authors:** Ahmet Ata Özçimen, Selma Ünal, Necmiye Canacankatan, Şerife Efsun Antmen

**Affiliations:** 1 Mersin University Faculty of Science and Art, Department of Biology, Mersin, Turkey; 2 Mersin University Faculty of Medicine, Department of Pediatrics, Division of Pediatric Hematology, Mersin, Turkey; 3 Mersin University Faculty of Pharmacy, Department of Biochemistry, Mersin, Turkey; 4 Mersin University Medical Services Vocational School, Mersin, Turkey

**Keywords:** Sickle cell disease, inflammation, Cytokine, TNFSF15 gene

## Abstract

**Objective:** The aim of this study was to investigate the relation between tumor necrosis factor-superfamily 15 (TNFSF15) gene expression and clinical findings in children with sickle cell disease (SCD).

**Materials and Methods:** Forty-nine patients with SCD and 38 healthy controls were included in this study. TNFSF15 gene expression and plasma levels were analyzed. TNFSF15 gene expression was compared in subgroups considering the frequency of painful crises and acute chest syndrome (ACS).

**Results:** It was found that TNFSF15 gene expression was significantly higher in patients with SCD than the controls (p=0.001), whereas there was no significant difference between the patients with SCD and the control groups considering plasma levels of TNFSF15. TNFSF15 gene expression was also significantly higher in SCD patients with ACS (p=0.008).

**Conclusion:** These findings suggest that TNFSF15 may have a role in the pathogenesis of SCD presenting with ACS. Further studies on larger groups are needed to determine the function of TNFSF15 in SCD patients with ACS and pulmonary hypertension. Analysis of TNFSF15 expression may also serve as a promising approach in ACS therapy.

## OZET

**Amaç:** Bu çalışmanın amacı, orak hücre hastalığı (OHH) olan çocukların klinik bulgularıyla birlikte tümör nekroz faktör süperailesi-15 (TNFSF15) gen ifadesi arasındaki ilişkinin araştırılmasıdır.

**Gereç ve Yöntemler:** Bu amaç için, OHH’li 49 hasta ve 38 sağlıklı kontrol bu çalışmaya dahil edilmiştir. TNFSF15 gen ifadesi ve plazma düzeyleri analiz edilmiştir. Aynı zamanda, TNFSF15 gen ifadesi akut göğüs ağrısı ve ağrılı kriz sıklığına göre de alt-gruplar karşılaştırılmıştır.

**Bulgular:** STNFSF15 plazma düzeyleri ile ilişkili olarak kontrol ve OHH’li hastalar arasında anlamalı bir fark bulunmazken, TNFSF15 gen ifadesi düzeyleri OHH hastalarında kontrol grubuna göre anlamlı bir şekilde artmış olarak bulunmuştur (p=0,001). TNFSF15 gen ifadesi aynı zamanda akut göğüs ağrısı olan OHH’li hastalarda anlamlı olarak artmış bulunmuştur (p=0,008).

**Sonuç:** Bu bulgular, TNFSF15 gen ifadesinin özellikle akut göğüs ağrılı OHH patogenezinde bir rolü olacağını düşündürmektedir. Pulmoner hipertansiyonlu ve akut göğüs ağrılı OHH hastalarında TNFSF15 işlevinin belirlenmesi için ileri geniş grup çalışmalarına ihtiyaç duyulmaktadır. TNFSF15 gen ifadesi aynı zamanda, OHH tedavisinde yeni bir yaklaşıma da katkı sağlayacaktır.

## INTRODUCTION

Sickle cell disease (SCD) is characterized by periodic vaso-occlusive crises, chronic hemolysis, and frequent infections that are accompanied by pain and organ damage [1,2]. Vaso-occlusive crises have been generally attributed to the abnormal shape and poor deformability of sickle erythrocytes [3]. Recent studies have pointed out that sickle red blood cells (SS RBCs) have the ability to bind vascular endothelial cells [4,5] and ultimately cause hypoxia and infarction. This increased adherence is directly related to the vaso-occlusive crises [6]. Vascular cell adhesion molecule-1 (VCAM-1) and intercellular adhesion molecule-1 (ICAM-1) are members of the immunoglobin superfamily. It was demonstrated that the expression of VCAM-1, ICAM-1, and E selectin were increased after incubation of SS RBCs with endothelial cells in vitro [4,7]. The interactions between these molecules are mediated by the activated endothelial cells and by inflammatory cytokines such as tumor necrosis factor-α (TNF-α) [8]. It was found that the adherence of SS RBCs is easier to TNF-α activated endothelial cell monolayers than to the resting endothelial cells [5,9].

The TNF and TNF receptor superfamilies (TNFRs) of proteins take part in the regulation of many important biological processes such as development, organogenesis, apoptosis, inflammation, and innate and adaptive immunity [10,11,12]. In humans, a total of 29 TNFRs and at least 18 specified members of the TNF superfamily with 15%-25% amino acid sequence homology have been identified [11,13]. TNF and IL-1α induce expression of an endothelial cell-derived TNF-like factor, tumor necrosis factor superfamily member 15 (TNFSF15), which has been recently discovered, and they also induce a ligand for death-domain receptor 3 (DR3) and decoy receptor 3 (DcR3) [14]. DR3 shows the highest homology to TNFR1. Interestingly, TNFR1 is ubiquitously expressed but DR3 is preferentially expressed by lymphocytes, which are efficiently induced after T cell activation [15]. There are several variants of TNFSF15. These have been discovered as the products of different transcripts generated with the use of cryptic splice sites and alternate exons. TNF superfamily ligand A (TL1A) is the longest and most abundant form of TNFSF15 [16].

In previous studies, it was reported that TNFSF15 took part in the development of diverse T cell-mediated autoimmune diseases, such as inflammatory bowel disease (IBD), and in experimental models such as chronic murine ileitis and autoimmune encephalomyelitis [17,18,19,20,21,22,23,24,25].

Although signal mediators in SCD were studied at the cellular and gene expression levels in many experimental studies, the effects of inflammatory cytokines in the pathogenesis of SCD remain to be studied [26].

The aim of our study is to determine the gene expression and the serum levels of TNFSF15 and investigate the relation between TNFSF15 gene expression and clinical findings in children with SCD. 

## MATERIALS AND METHODS

**Study Population**

We investigated TNFSF15 gene expression and serum levels in children with SCD. The diagnosis of SCD was confirmed according to hematological and clinical data. Forty-nine children (34 males and 15 females; mean age: 9.24±3.66 years, range: 3-17 years) with SCD (38 SS, 11 Sβ), who were followed in the Pediatric Hematology Unit of Mersin University, Mersin, Turkey, were included in the study (Group 1: patients with SCD). Thirty-eight children (19 males and 19 females; mean age: 7.84±3.53 years, range: 2-16 years) were consecutively selected from healthy unrelated subjects from the same geographic area of Turkey (Group 2; controls).

The information about painful crisis, acute chest syndrome (ACS), and stroke was taken from hospital medical records.

Patients who had histories of vaso-occlusive crises and erythrocyte transfusion in the last 1 month were excluded.

The patients were classified according to history of painful crises in the last 1 year. Group 1a included patients who had no painful crises, Group 1b included patients who had 1-4 painful crises, Group 1c included patients who had 5-10 painful crises, and Group 1d included patients who had more than 10 painful crises in the last 1 year. The patients were also classified according to ACS data.

The protocol of this study was approved by the Ethics Committee of the School of Medicine of Mersin University. The investigation conforms to the principles outlined in the Declaration of Helsinki.

**RNA Isolation and Quantitative Real-Time PCR**

Blood samples were collected from patients with SCD who had been treated at Mersin University Research Hospital and from healthy subjects as controls. The blood mononuclear cells were separated by Red Blood Cell Lysis Buffer (Roche, Mannheim, Germany). Total RNA was extracted from peripheral blood mononuclear cells using the High Pure RNA Isolation Kit (Roche) following the manufacturer’s protocol including an additional genomic DNA digestion step with DNase I.

Five micrograms of total RNA was used to synthesize cDNA. First-strand cDNA synthesis was conducted using the Transcriptor First Strand cDNA Synthesis Kit (Roche) according to the manufacturer’s recommendations. Real-time monitoring of PCR reactions was performed with the LightCycler®2.0 Real-Time PCR Detection System (Roche). Each reaction was conducted with TNFSF15-specific primers. The specific PCR assay was designed with the web-based Probe Finder software, using primers and probes from the Universal Probe Library (www.roche-applied-science.com/sis/rtpcr/upl/index.jsp, UPL, Roche). We selected a UPL assay for the TNFSF15 real-time PCR with the following primer-probe combination: forward primer 5′- caagggcacacctgacagt -3′ and reverse primer 5′- cctagttcatgttcccagtgc- 3′, with UPL probe #33 (Cat. No. 04687663001, Roche). β-Actin (ACTB) was used as an internal control (Cat. No. 0504616500, UPL Human ACTB Gene Assay, Roche).

**Determination of Serum TNFSF15**

Serum TNFSF15 levels were determined by ELISA assay by pairing a monoclonal antibody with biotinylated anti-TNFSF15. A monoclonal antibody specific for hTL1A was coated onto the wells of the microtiter plate. hTL1A was recognized by the addition of a biotinylated monoclonal antibody specific for hTL1A. After removal of excess biotinylated antibody, streptavidin-peroxidase was added. Peroxidase activity was quantified using the substrate 3,3’,5,5’-tetramethylbenzidine. Plates were read in an ELISA plate reader at 450 nm after acidification. The intensity of the color reaction is directly proportional to the concentration of hTL1A in the samples [Manual TL1A, Soluble (Human) Detection Set for ELISA Application, Apotech, Epalinges, Switzerland].

**Statistical Analysis**

Statistical analyses were carried out using SPSS 11.5 and STATISTICA 6.0. Since the data were distributed nonnormally, the Kruskal–Wallis test, the Mann–Whitney U test, and Spearman’s correlation analysis were used in comparisons between groups. A value of p<0.05 was considered to represent a statistically significant result.

## RESULTS

**Figure 1:**
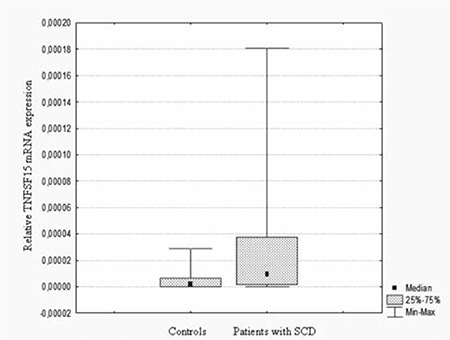
The relative expression of TNFSF15 gene in patients with SCD (n=49) and the control group (n=38) (p=0.001).

**Figure 2:**
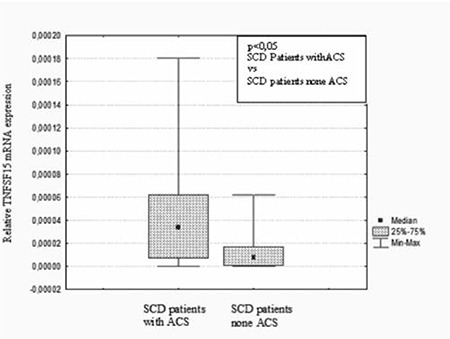
The relative expression of TNFSF15 gene in SCD patients with ACS (n=16) and in SCD patients without ACS (n=33) (p=0.008).

Forty-nine patients with SCD and 38 healthy controls were included in this study. The mean TNFSF15 gene expressions were 0.24±0.35 and 0.048±0.07 in the patients with SCD and the controls, respectively. As shown in Figure 1, TNFSF15 gene expression was significantly higher in patients with SCD than the controls (p=0.001). On the other hand, there was no difference between the patients with SCD and the controls in terms of plasma levels of TNFSF15 (Table 1). As we correlated TNFSF15 gene expression and plasma level, a negative nonlinear relation (r=-0.037, p=0.797) in patients and a positive nonlinear relation (r=0.312, p=0.05) in the control group were found. We also compared TNFSF15 gene expression in subgroups according to frequency of painful crises, history of ACS, and stroke. According to history of painful crises in the last 1 year, 11 patients in Group 1a, 21 patients in Group 1b, 7 patients in Group 1c, and 10 patients in Group 1d were included. Sixteen patients (33%) had ACS and 4 (8%) patients had stroke. There were no differences between the groups according to frequency of painful crises in terms of TNFSF15 gene expression and plasma levels (p>0.05) (Table 2). Interestingly, SCD patients with ACS showed much higher TNFSF15 levels (p=0.008). TNFSF15 gene expression was also significantly higher in SCD patients with ACS (p=0.008) (Figure 2). Mean TNFSF15 gene expressions were 0.46±0.50 and 0.14±0.18 in SCD patients with and without ACS, respectively (Table 2). We also examined the TNFSF15 gene expression and serum levels in the SCD patients with and without stroke and did not find a significant difference (p>0.05).

## DISCUSSION

In this study, we aimed to investigate the relation between the gene expression and serum levels of TNFSF15 and clinical findings in children with SCD.

TNFSF15 is the gene encoding antiangiogenic protein expressed abundantly on endothelial cells, but not on B or T cells. Additionally, the expression of this protein is inducible by TNF and IL-1α. This cytokine inhibits endothelial cell proliferation and thus may function as an angiogenesis inhibitor [13].

TNFSF15 gene expression levels were investigated in various inflammatory diseases [17,18,19,20,21,22,23,24,25]. The studies that investigated TNFSF15 were experimental and the first clinical study with TNFSF15 was carried out in patients with IBD. It was reported that TNFSF15 polymorphisms are associated with susceptibility to IBD in a European cohort [18]. Additionally, TNFSF15 single nucleotide polymorphisms and haplotypes were found to be strongly correlated to Crohn’s disease in Japanese patients [17,19]. The association was confirmed by studies involving 2 European IBD cohorts [18,20,21].

Furthermore, Al-Lamki et al. suggested that TNFSF15 might contribute to renal inflammation and injury through DR3-mediated activation of NF-κB and caspase-3 [22]. In recent studies it was demonstrated that, due to inflammation, TNFSF15 gene expression levels could be varied. Additionally, alterations in TNFSF15 levels from T-cell lymphocytes were reported [22,23,24,25].

In this study, we evaluated both the gene expression and the serum levels of TNFSF15 in children with SCD and we found that TNFSF15 gene expression was significantly higher in patients with SCD than the controls (p=0.001). Acute pain crises in SCD are usually accompanied with infection and/or inflammation, with IL 1-β and TNF-α released [27,28,29]. Thus, we assumed that TNFSF15 gene expression and serum levels would be highest in Group 1d, in which the frequency of painful crises was high, but there were no differences between groups. It is supposed that TNFSF15 gene expression may have not a role by itself in the pathogenesis of painful crises. Additionally, contrary to expectations, we could not find any difference related to serum levels of TNFSF15 between the patients with SCD and the control group. As we correlated the groups separately, we found a negative nonlinear relation (r=-0.037, p=0.797) in patients and a positive nonlinear relation (r=0.312, p=0.05) in the control group between TNFSF15 gene expression and plasma level. The low abundance of TNFSF15 protein in SCD patients might therefore be involved in the posttranslational unknown modifications of late conversion of TNFSF15 protein.

Interestingly, TNFSF15 gene expression and serum level were dramatically higher in SCD patients with ACS than in the other patients (p=0.008). It may be suggested that TNFSF15 may have a role in the pathogenesis of ACS. Similar to our results, Safaya et al. also found marked expression of TNFSF15 and DR3 in human pulmonary arterial and lung microvascular endothelial cells by induction of butyrate and reported that TNFSF15 might modulate inflammation and sickle cell vasculopathy [30]. According to our results, we suggest that TNFSF15 gene expression might be altered in pulmonary endothelial cells in patients with ACS, and this may lead to the activation of other proinflammatory cytokines, which causes clinical symptoms of SCD.

Pulmonary hypertension is one of the most important complications in patients with SCD in older age. Endothelial cell damage and inflammation have a prominent role in the pathogenesis of these complications, as in ACS. Thus, it might be expected that TNFSF15 gene expression has increased in patients with pulmonary hypertension. Since there was no patient with SCD and pulmonary hypertension in our study population, we could not research the relation between the TNFSF15 gene and pulmonary hypertension. 

In summary, it might be suggested that increased TNFSF15 gene expression may have a role in the pathogenesis of the chronic inflammatory status of SCD patients. The higher antiangiogenic TNFSF15 gene expression levels may contribute to chronic hypoxia status, development of organ damage, and ACS in these patients. The present study is limited by its number of patients. The mechanisms should be further tested by in vivo models. Further studies on larger groups are needed in order to appreciate the function of TNFSF15 in SCD patients with ACS and pulmonary hypertension. TNFSF15 expression may also serve as a promising approach in ACS therapy.

**Acknowledgments**

This study was supported by the Scientific and Technological Research Council of Turkey [TÜBİTAK, SBAG-HD-255 (107S308)]. The authors express their appreciation to Prof. Dr. Yasemin Kaçar for her valuable contributions in editing the manuscript and to Assoc. Prof. Bahar Taşdelen for her valuable assistance in statistical evaluation. 

**Conflict of Interest Statement**

The authors of this paper have no conflicts of interest, including specific financial interests, relationships, and/ or affiliations relevant to the subject matter or materials included.

## Figures and Tables

**Table 1:**
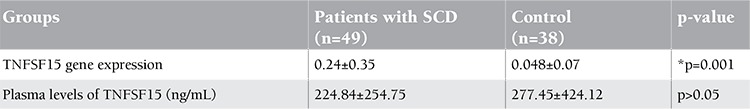
TNFSF15 gene expression and plasma levels of SCD patients and controls.

**Table 2:**
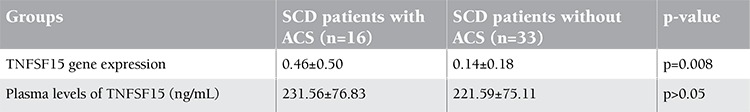
TNFSF15 gene expression and plasma levels of SCD patients with ACS and without ACS.
